# Self-organization and circular causation as synergetic foundations of social interaction: a multilayer network perspective

**DOI:** 10.3389/fnetp.2026.1776969

**Published:** 2026-04-23

**Authors:** Viktor Müller

**Affiliations:** Center for Lifespan Psychology, Max Planck Institute for Human Development, Berlin, Germany

**Keywords:** circular causation, hyper-brain network dynamics, multilayer networks, network physiology, self-organization, social interaction, within- and cross-frequency coupling

## Abstract

Social interaction emerges from complex, dynamic couplings among neural, physiological, and behavioral subsystems across individuals. Here, we propose the *Synergetic Multilayer Social Interaction Hypothesis*, which frames interpersonal coordination as a self-organizing process in multilayer networks, where circular causation across levels gives rise to emergent order parameters. These order parameters—dynamical, capturing temporal patterns such as shared oscillations and phase relationships, and structural, reflecting stable network topologies or hyper-brain modules—serve as measurable markers of collective organization. Empirical examples from hyperscanning, ensemble music performance, choral singing, and romantic interactions demonstrate how local interactions within and between subsystems or brains stabilize macro-level coordination patterns, providing preliminary support for the framework. The hypothesis predicts that stronger and more symmetrical coupling across neural, autonomic, and/or behavioral layers will produce more robust and persistent alignment between interacting individuals. This framework integrates network neuroscience, dynamical systems theory, and synergetics, offering a novel conceptual and methodological roadmap for investigating the mechanisms of social cognition and collective behavior.

## Introduction

As figuratively expressed by Henri Poincaré in his well-known book “Science and Hypothesis”, “the aim of science is not things themselves, as the dogmatists in their simplicity imagine, but the relations between things; outside those relations there is no reality knowable” (Poincaré, 1905, p. xxi). Alexander von Humboldt, who conceived the universe in its unity and dynamic interrelation, where everything is related to everything else, noted in his “Diaries of the Voyage to America” (“Tagebücher der Amerikanischen Reise”): “Everything is interaction” (“Alles ist Wechselwirkung,” von Humboldt, cited in Ette, 2018; see also http://resolver.staatsbibliothek-berlin.de/SBB0001527C00000000, p. 41). These ideas are consistent with Hermann Haken’s approach to synergetics: “The word “synergetics” is composed of two Greek words and means “working together”. In many disciplines, ranging from astrophysics over biology to sociology, we observe that very often the cooperation of many individual parts of a system leads to macroscopic structures or functionings” (Haken, 1977, p. 2).

In line with synergetics and dynamic systems theory, complex natural phenomena are best understood as emerging from the interactions among multiple subsystems (Forrester, 1968; Haken, 1983; 1984). When considered in isolation, each subsystem may follow relatively simple intrinsic dynamics; however, the collective behavior of the overall system arises from coupling mechanisms operating within and between these subsystems. These interactions give rise to a higher-order or *superordinate* system that constrains and organizes the dynamics of its components by imposing boundary conditions on their activity (Buzsáki, 2006; Haken, 2006; 2016; Noble, 2012; Haken and Portugali, 2016; 2021; Müller et al., 2018a; 2019a; 2021; Müller, 2022; Delius and Müller, 2023).

In biological studies of collective behavior, interactions among individual organisms—and between them and their environment—can give rise to coordinated swarm behavior that is often described as a “superorganism” (Trianni et al., 2011). The concept was first proposed by the American entomologist Wheeler (1911) in his seminal work “The ant-colony as an organism” (*cf.* Hoffecker, 2013), emphasizing that the colony functions as an integrated whole capable of collective decision-making. Similar principles are observed in other animal societies, such as fish schools, bird flocks, mammalian packs or herds, and even human groups engaged in coordinated activities (Detrain and Deneubourg, 2006; Gelblum et al., 2015; Müller et al., 2021; Müller, 2022). These systems or societies exemplify *self-organizing* and *auto-regulating* behavior. Self-organization refers to processes in which spontaneous order or spatiotemporal patterns emerge from local interactions among parts of an initially disordered system, often initiated by random fluctuations and amplified by positive feedback. The resulting pattern is an *emergent property*, arising from interactions among system components rather than from any single element alone (Haken, 1983; 1984; 2006; Haken and Portugali, 2016; 2021).

As emphasized by Ilya Prigogine and colleagues, self-organization typically occurs in nonlinear systems far from thermodynamic equilibrium, where fluctuations can drive the emergence of order (Nicolis and Prigogine, 1977; Prigogine and Stengers, 1984). Ashby (1952) similarly suggested that such systems tend to evolve toward stable equilibrium states or attractors in phase space. Within this framework, a superordinate system—or superorganism—represents a higher-order structure that both emerges from and constrains the dynamics of its components or constituents. This recursive interplay exemplifies *circular causation*, in which bottom-up interactions among components give rise to emergent structures that in turn exert top-down influence, stabilizing and regulating the system as a whole.

Analogous principles apply to social systems and behavior. Human social life crucially depends on relations between individuals—that is, on the continuous coordination of perception and action in time and space. In everyday situations, people must align their behaviors, gestures, and intentions with those of others to achieve shared or complementary goals (Sänger et al., 2011; Keller et al., 2014; Müller et al., 2021). Such interpersonal coordination not only facilitates joint action but also supports the emergence of congruent intentions and shared understanding, which are central to social behavior, although not always required for transient alignment of minds and bodies (Gallotti and Frith, 2013; Keller et al., 2014; Gallotti et al., 2017; Dumas et al., 2020; Müller et al., 2021; Keller, 2023). As Hasson and Frith (2016), p. 2) aptly put it, “…interactions with other members of a group can fundamentally shape the way we behave in the world, and alignment is a ubiquitous feature of such interactions.” This alignment presupposes multiple forms of synchronization and temporal (and often spatial) coordination across interacting systems or subsystems—whether neural, physiological, or behavioral in nature.

From a systems perspective, such interpersonal alignment can be conceived as a process of *self-organization* within and across multiple layers of interacting subsystems. Neural, physiological, and behavioral processes each constitute relatively autonomous dynamical systems, yet they are continuously coupled through reciprocal influences that form *multilayer networks* (*MLNs*) within and between individuals. Within this framework, *circular causation*—the bidirectional interplay between lower-level component dynamics and higher-order collective patterns—plays a decisive role in shaping coordinated social behavior. On the one hand, local fluctuations and individual actions can amplify or suppress emergent group-level patterns (upward causation); on the other hand, these emergent patterns exert constraining influences on the participating subsystems, stabilizing or reorganizing their activity (downward causation). Such recursive interactions give rise to adaptive and coherent interpersonal dynamics that can be captured using MLN models and synergetic principles, offering a unifying account of how complex social interaction emerges from interdependent neural, physiological, and behavioral processes.

Empirically, this framework can be operationalized by quantifying the dynamic interactions within and between individuals across multiple physiological and behavioral layers. Neural synchronization can be assessed through measures of intra- and inter-brain coupling derived from EEG or other neuroimaging (e.g., fMRI, fNIRS) recordings, while autonomic coordination can be captured by analyzing respiration, heart rate variability (HRV), muscle activity, and other physiological signals using cross-recurrence or coherence methods. Behavioral alignment, in turn, can be studied through temporal coordination of voice, facial expression, or movement patterns. Integrating these data streams within an *MLN model* allows for the examination of cross-level dependencies and mutual influences among subsystems. Importantly, metrics of *circular causation*, such as bidirectional information flow or mutual prediction between layers, provide a direct means to test how bottom-up and top-down interactions contribute to the emergence and stability of interpersonal synchrony.

The present article aims to provide a conceptual review of synergetic principles, interpersonal synchrony, and MLN dynamics with regard to social interaction. Building on these foundations, a *Synergetic Multilayer Social Interaction Hypothesis* (*SMSIH*) is proposed. Investigating the hyper-brain networks in their close connections with physiological systems and subsystems is crucial for understanding the mechanisms of social interaction and interpersonal action coordination. Finally, ways of proving the *SMSIH* will be provided and discussed.

The present framework is closely related to the emerging field of Network Physiology, which investigates how physiological systems interact dynamically to produce integrated function across multiple scales (Bashan et al., 2012; Faes et al., 2014; Bartsch et al., 2015; Liu et al., 2015; Ivanov et al., 2016; Rizzo et al., 2020; Ivanov, 2021). While Network Physiology has primarily focused on interactions within the organism, the present work extends this perspective to interacting individuals by conceptualizing social systems as multilayer networks of coupled physiological and neural processes. In this sense, the proposed *SMSIH* can be understood as a natural extension of Network Physiology to the domain of social interaction.

## Theoretical foundations

### Self-organization as a synergetic principle in social interaction dynamics

As mentioned above, *self-organization* refers to the spontaneous emergence of ordered structures or patterns in systems composed of many interacting elements, without the need for centralized control (Haken, 1983; 1984; 2006; Haken and Wunderlin, 1991; Haken and Portugali, 2016; 2021). When a system is driven far from equilibrium, small fluctuations in local interactions can be amplified through positive feedback loops, leading to the formation of macroscopic order parameters that constrain and coordinate the behavior of individual components (Nicolis and Prigogine, 1977; Prigogine and Stengers, 1984). This principle can be observed not only in physical and biological systems but also in social contexts, where coordination among individuals gives rise to collective patterns of behavior (Kelso, 1995; Tognoli et al., 2018; 2020; Delius and Müller, 2023).

A compelling everyday example of social self-organization is synchronized clapping in audiences. As shown by Néda and colleagues (Néda et al., 2000a; 2000b), the emergence of rhythmic coordination during applause illustrates a spontaneous transition from incoherent to coherent collective behavior. When clapping occurs rapidly and irregularly, synchronization is hindered due to large variability in individual clapping frequencies. However, as individuals spontaneously slow down—effectively doubling the period of their clapping—the variability decreases, and synchronization becomes possible. This *phase transition* between desynchronized and synchronized states exemplifies how collective order can emerge through mutual adaptation among individuals, even in the absence of external coordination. Yet, as the audience attempts to increase the overall intensity of applause, the collective rhythm destabilizes and the system reverts to a disordered, high-energy state. This dynamic trade-off between maximal noise intensity and optimal synchrony reflects a *self-organizing principle of energetic optimization*, consistent with the *free-energy principle* proposed in theoretical neuroscience: systems tend to minimize free energy by maintaining a balance between stability and variability in interaction with their environment (Friston et al., 2006; 2025; Friston, 2010; Bruineberg et al., 2018; Palacios et al., 2019). It also shows the way self-organization may occur and illustrates the emergence of order from chaos or enhanced fluctuations in social interaction dynamics (Prigogine and Stengers, 1984; Haken, 2006; Haken and Portugali, 2016; 2021).

Beyond its energetic and dynamic aspects, the applause scenario also demonstrates the inherently *rhythmic* nature of social coordination. When individuals begin to clap, their rhythms initially compete, but very quickly a shared tempo emerges—a collective rhythm that is jointly negotiated and dynamically maintained (Kriz, 2001). Rhythm thus serves as a fundamental *organizing principle* not only in music, speech, and dance (Brown, 2018; Kotz et al., 2018; Savage et al., 2021), but also in a wide range of cultural and aesthetic domains, including architecture, poetry, theater, and visual arts (Chan, 2012; Cureton, 2015; Hopsch and Lilja, 2017; Thapa, 2017; Mohamed, 2018; Levin et al., 2019; Abdulhussain et al., 2023). From a synergetic perspective, rhythmic coordination represents a temporal mode of *interpersonal coupling*, through which individuals align perception and action across multiple layers—neural, physiological, and behavioral. In this sense, rhythm constitutes a *universal anthropological principle* that organizes human experience in time and space and forms the foundation for the emergence of higher-order social coherence (Iberall and McCulloch, 1968; 1969; Warner et al., 1987; Warner, 1988; Jin et al., 2024; Shen et al., 2025).

Another striking example of social self-organization is the spontaneous formation of lanes in pedestrian crowds. When two groups of people move in opposite directions in a confined space—such as a busy street, corridor, or train station—individuals do not require central coordination or explicit rules to avoid collisions. Instead, global order emerges spontaneously from local interactions among individuals, who continuously adjust their trajectories and velocities based on the perceived movements of others (Helbing and Molnár, 1995; Helbing et al., 1997; 2000; 2001; 2005; Moussaïd et al., 2010; 2011). Over time, these local adaptations lead to the formation of stable unidirectional lanes, allowing smoother flow and minimizing mutual interference. This phenomenon demonstrates that complex collective patterns can emerge from simple, local behavioral rules (e.g., pedestrians adjust their speed and direction, maintain personal space, and respond to nearby motion cues). Through repeated micro-interactions, these local adjustments generate *global coherence* and *stable flow structures*, all without centralized control or explicit communication.

**FIGURE 1:**
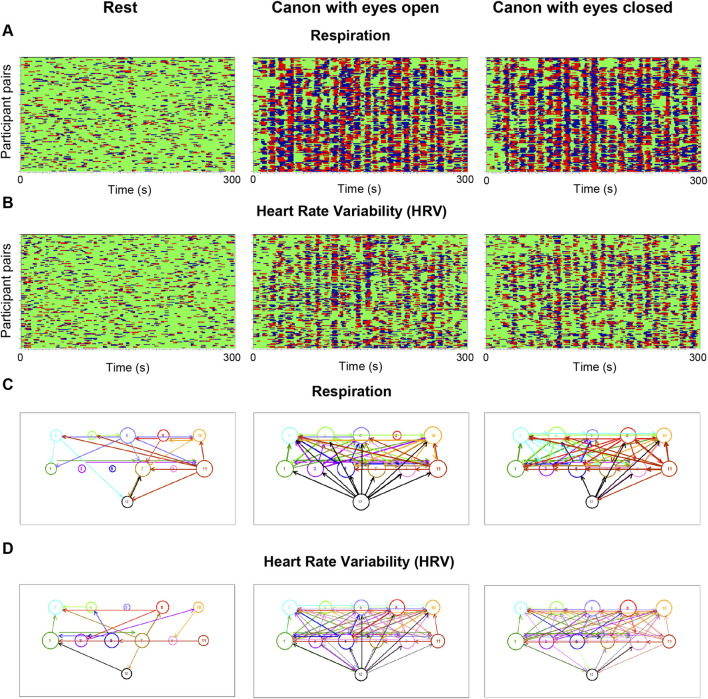
Phase synchronization and connectivity patterns of the choir for respiration and heart rate variability (HRV) during rest and canon singing. **(A)** Phase synchronization patterns for respiration. Pairwise phase synchronization of the entire choir (132 subject pairs) over time (300 s) is shown for rest (left column), canon singing with eyes open (middle column), and canon singing with eyes closed (right column). Each horizontal line represents one pair of participants. For each participant, 11 lines depict the coded phase difference (Δφ) between that participant and all other choir members. Phase relations are color-coded as follows: −π/4 < Δφ < 0 (blue), 0 < Δφ < +π/4 (red), and Δφ < −π/4 or Δφ > +π/4 (green; non-synchronized). All diagrams depict phase synchronization at 0.24 Hz. **(B)** Phase synchronization patterns for HRV. The structure and color coding are identical to those in **(A)**, and synchronization is shown at the same frequency (0.24 Hz). **(C)** Connectivity structure of respiration. Directed networks are derived using the *Integrative Coupling Index* (*ICI*), computed from the phase differences (Δφ) shown in **(A)**, for rest, canon singing with eyes open, and canon singing with eyes closed. Nodes represent choir members (1–11: singers; 12: conductor), with node size proportional to the total number of incoming and outgoing connections. Link thickness reflects coupling strength, and arrows indicate the direction of causal influence. Notably, connections from the conductor to the choir members are predominantly outgoing, indicating that changes in respiratory phase emerge earlier in the conductor and are subsequently transmitted to the choir. **(D)** Connectivity structure of HRV. Directed networks are constructed analogously to **(C)**, based on *ICI* values derived from the HRV phase synchronization patterns shown in **(B)** Panels A and B are modified from Figure 3 in Müller and Lindenberger (2011) under a CC BY 4.0 license, and Panels C and D are modified from Figure 4 in Müller and Lindenberger (2011), also under a CC BY 4.0 license.

A further illustrative example of social self-organization in coordinated social behavior is provided by choral singing (Müller and Lindenberger, 2011). Figure 1 illustrates synchronization patterns of respiratory and cardiac (HRV) rhythms across choir members during rest and during canon singing with eyes open and eyes closed. During rest, synchronization exhibits a largely sporadic and weakly structured pattern, consistent with predominantly independent oscillatory dynamics across individuals. In contrast, during singing—both with visual access to the conductor or other choir members (eyes open) and without it (eyes closed)—the synchronization patterns become highly structured, showing strong and persistent phase alignment among choir members. The corresponding connectivity diagrams further reveal that the conductor’s influence is predominantly unidirectional, from the conductor to the choir members. This indicates that changes in the oscillatory activity of respiration (and to some extent also HRV) emerge earlier in the conductor and are subsequently transmitted to the choir, in line with the conductor’s functional role in shaping collective timing and coordination (Müller and Lindenberger, 2011; Delius and Müller, 2023). From a synergetic perspective, the transition from resting state to collective singing reflects a process of social self-organization, in which initially weakly coupled individual oscillators become dynamically entrained into a coherent macroscopic pattern. Within this framework, the conductor can be interpreted as a control parameter that biases the system toward a coordinated regime, facilitating the emergence and stabilization of collective order without prescribing the detailed dynamics of individual choir members.

### Enslaving principle, order parameters, and circular causality as emergent properties of social interaction

As illustrated, complex systems self-organize in the way that a few order parameters emerge from the collective dynamics of many interacting components. Once established, these order parameters “*enslave*” or constrain the behavior of the components that generated them, resulting in *circular causality*: the parts create the global pattern, and the global pattern, in turn, shapes the behavior of the parts. This recursive relationship gives rise to emergent higher-level order and coordinated system behavior. As Haken (1996, p. 43) vividly described: “As it turns out, by their collective action the individual parts, or puppets, themselves act on the order parameters, i.e., on the puppeteers. While on the one hand the puppeteers (order parameters) determine the motion of the individual parts, the individual parts in turn determine the action of the order parameters. This phenomenon is called circular causality. The principle of circular causality allows us to interpret the slaving principle in yet another fashion. Because the individual parts of the system determine or even generate the order parameters which in turn enslave the individual parts, the latter determine their behavior cooperatively. It is tempting to describe this phenomenon in anthropomorphic terms as consensus finding by the individual parts. Thus enslavement and consensus finding are two sides of the same coin.”

In previous cases with synchronized applause and lane formation in pedestrian crowds, the transition from disordered individual behavior to coherent collective dynamics reflects the emergence of an order parameter—a macroscopic variable that captures the dominant mode of coordination within the system. During applause, for instance, the collective rhythm that emerges after a period-doubling transition serves as an order parameter that constrains individual clapping rates, aligning them to the shared tempo (Néda et al., 2000a; 2000b). Similarly, in pedestrian flows, the appearance of unidirectional lanes represents an emergent global structure that regulates how individuals adjust their trajectories to maintain smooth passage (Helbing and Molnár, 1995; Helbing et al., 1997; 2000; 2001; 2005; Moussaïd et al., 2010; 2011). In both scenarios, the order parameter “enslaves” the micro-level behavior of the individual agents by stabilizing collective dynamics, while at the same time being continuously shaped by those very interactions—a hallmark of circular causality. Thus, local fluctuations and adaptive corrections among individuals give rise to a coherent macroscopic pattern, which in turn channels subsequent individual behavior. This bidirectional influences or ascendancies between micro- and macro-dynamics exemplifies how social self-organization can be understood in terms of synergetic principles: consensus formation, synchronization, and coordinated movement all emerge through the reciprocal interplay between parts and wholes. Crucially, these emergent macroscopic features are not reducible to micro-level descriptions of individual components but reflect new organizational properties of the system as a whole (Haken, 1983; 1984; Jirsa et al., 1994; Jirsa and Haken, 1996).

Buzsáki emphasized that in line with Haken’s principles of synergetics, “…emergence through self-organization has two directions. The upward direction is the local-to-global causation, through which novel dynamics emerge. The downward direction is a global-to-local determination, whereby a global order parameter ‘enslaves’ the constituents and effectively governs local interactions. There is no supervisor or agent that causes order; the system is self-organized. The spooky thing here, of course, is that while the parts do cause the behavior of the whole, the behavior of the whole also constrains the behavior of its parts according to a majority rule; it is a case of circular causation. Crucially, the cause is not one or the other but is embedded in the configuration of relations” (Buzsáki, 2006, p. 14; see also Haken, 1983; 2006).

A compelling illustration of circular causation and other synergetic principles in social coordination can be found in the context of choral singing (Delius and Müller, 2023). Within a choir, each singer contributes individually to the emergent collective sound through their vocal production or singing, thereby influencing the overall acoustic and temporal structure of the ensemble—an instance of *upward causation*. At the same time, the choir as a superordinate system or superorganism exerts *downward causation* by imposing global constraints on each singer, such as the shared tempo, pitch, and phrasing that define the ensemble’s coherence (Müller and Lindenberger, 2011; Müller et al., 2018a; 2019a; 2021; 2026; Müller, 2022; Delius and Müller, 2023). These reciprocal influences unfold not only at the level of voice production but also across multiple physiological subsystems—respiratory, cardiac, and neural—each dynamically coupled within and between individuals. Through these multilayer interactions, the choir self-organizes into a coherent, adaptive whole, exemplifying synergetic circular causation across hierarchical levels. From this perspective, the observed synchronization patterns and network configurations constitute emergent order parameters that capture the macroscopic state of collective coordination and constrain individual subsystem dynamics.

Importantly, while the conductor modulates the control parameters that bias the system toward coordination, the emergent order parameters themselves arise through self-organized interactions among choir members and persist even when direct visual control is reduced (eyes closed). This dissociation underscores the distinction between externally modulated control parameters and internally generated order parameters governing collective social behavior. Accordingly, the collective state of the choir both emerges from and regulates the activity of its members, illustrating how circular causality underlies the self-organized coordination of complex social systems (Delius and Müller, 2023).

## Multilayer network framework

### Multilayer network approach as a conceptual framework for social interaction

From a mathematical standpoint, complex systems can be represented as networks or graphs in which interacting elements (nodes) are connected by links indicating structural or functional relationships. The corresponding adjacency matrix *A* encodes this connectivity, where each entry *a*_*ij*_ specifies the connection from node *i* to node *j* such that *a*_*ij*_ = 1 if there is an edge between these nodes (or *w* if the link has an associated weight) or *a*_*ij*_ = 0 otherwise. Already in the late 1960s, pioneering work across biology, sociology, and physics emphasized that such networks of interacting units—from molecular assemblies to social groups—exhibit *emergent properties* that cannot be explained by the behavior of individual components in isolation (Forrester, 1968; Haken, 1983; 1984).

Complexity in living and social systems is characterized not only by hierarchical organization but also by multiplexity and interdependence. Each subsystem—be it cellular, neural, behavioral, or social—can itself be viewed as a network embedded within a larger network of networks. Nature thus favors systems of systems, in which interrelations across levels cannot be reduced to or aggregated within a single layer. Molecules interact to form cells, cells form tissues, tissues form organs, and organs form organisms; analogously, organisms interact to form groups, societies, and ecosystems—each level exhibiting self-organization and circular causation (De Domenico, 2022).

To formalize such interconnected multilevel dynamics, the *MLN framework* extends classical graph theory by adding a layer dimension. Each layer α represents a distinct domain or modality (e.g., neural, autonomic, behavioral), and a node *i* in layer α can be connected to any node *j* in the same or any other layer *β*. Connections within the same layer define *intralayer links*, while those across layers define *interlayer links*. Mathematically, these relationships are captured by the *multilayer adjacency tensor* $M_{j\beta }^{i\alpha }$ , a rank-4 tensor that encodes all possible intra- and interlayer connections among nodes (De Domenico, 2022).

Empirically, the MLN framework can be operationalized by integrating time-resolved measures from multiple physiological and behavioral domains. The corresponding formal definitions are given in Equations 1–9. Formally, the elements of the multilayer adjacency tensor can be defined as

$$M_{j\beta }^{i\alpha }\left(t\right)=C\left(x_{i}^{\alpha }\left(t\right),x_{j}^{\beta }\left(t\right)\right),$$ (1)

where $x_{i}^{\alpha }\left(t\right)$ denotes the time series recorded from node i in layer α , $x_{j}^{\beta }\left(t\right)$ denotes the time series recorded from node j in layer β , and $C\left(\cdot ,\cdot \right)$ represents a coupling function quantifying functional interaction between the signals (e.g., coherence, phase-locking value, transfer entropy, or recurrence-based measures) both within and between layers.

**FIGURE 2:**
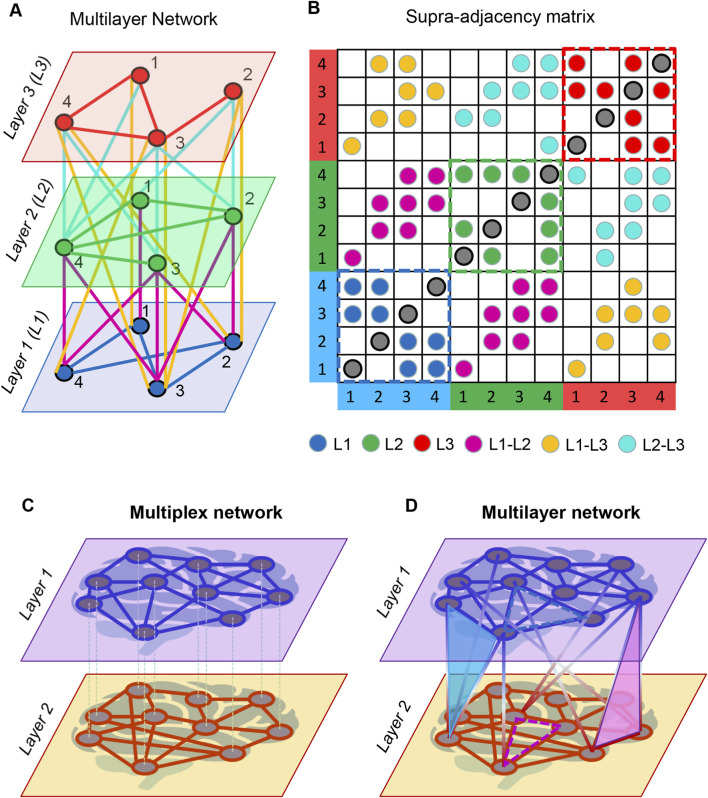
Schematic representation of multilayer networks (MLNs). **(A)** A three-layer MLN comprising four nodes per layer. Layers, nodes, intralayer links, and interlayer links are color-coded for clarity. **(B)** Supra-adjacency matrix corresponding to the MLN in **(A)**. Each circle represents a specific connection in the network. Intralayer connections appear in the diagonal blocks (indicated by dashed squares), whereas interlayer connections occupy the off-diagonal blocks, reflecting couplings between layers. **(C)** A two-layer single-brain *multiplex network*. Each node has a replica in every layer, and each layer represents a different type of connectivity (e.g., distinct EEG frequency bands, structural vs. functional connectivity, or cell-signaling pathways). Edges may differ across layers, but interlayer connections are typically either absent or restricted to links between corresponding replica nodes. **(D)** A two-layer single-brain *multilayer* network. Here, nodes may be connected both within and across layers, and interlayer connections are not restricted to replica nodes; any node in one layer may link to any node in another. In a hyper-frequency network (HFN), intralayer connections correspond to WFC, while interlayer connections correspond to CFC. Examples of higher-order (three-body) dependencies are shown: dashed blue and red triangles represent dependencies within layers, whereas solid blue and red triangles illustrate dependencies spanning across layers. The red triangle shows two nearest neighbors of a target node in layer one that are directly connected in layer 2; the blue triangle shows the converse case. Panels A and B modified from Figure 3 in Müller et al. (2026) under a CC BY 4.0 license, and Panels C and D modified from Figures 7A,B in Müller (2022), also under a CC BY 4.0 license.

Although this tensorial representation is conceptually elegant, its high dimensionality poses computational challenges. To manage this complexity, one can apply *matricization*, which flattens the tensor into a lower-rank object known as the *supra-adjacency matrix*. If a system comprises *N* nodes and *L* layers, $M_{j\beta }^{i\alpha }$ spans a space of N × N × L × L dimensions, while its flattened supra-adjacency matrix is defined in a (NL) × (NL) space. In this representation, *intralayer adjacency matrices* occupy the diagonal blocks, and *interlayer adjacency matrices* occupy the off-diagonal blocks (see Figures 2A,B for details). This configuration preserves the full informational content of the original tensor but allows one to apply standard linear algebraic and network analytic tools—provided that the interpretative distinction between layers and nodes is maintained (Boccaletti et al., 2014; Kivelä et al., 2014; De Domenico, 2022; 2023).

Complex networks can be described as multiplex or multilayer networks that exhibit a characteristic multidimensional or multilayered organization (De Domenico et al., 2013; 2015; 2016; Boccaletti et al., 2014; Kivelä et al., 2014; De Domenico, 2017; Pilosof et al., 2017; de Arruda et al., 2018). Multiplex networks constitute a particular class of MLNs in which the same set of nodes is replicated across all layers, with each layer representing a distinct type of relation among the same entities (e.g., multimodal brain networks derived from MRI, DTI, EEG, or MEG). In such networks, intralayer edges link nodes within each modality, and interlayer connections—if present—typically occur only between replica nodes across layers.

In contrast, the broader MLN framework does not require that all nodes appear in every layer and allows for rich patterns of interlayer connectivity. Layers may differ in their node sets, the nature of their edges, or the type of coupling between layers. These distinctions are illustrated in Figures 2C,D. MLN modelling expands the analytical possibilities for characterizing network topology and function, enabling flexible combinations of layers and customized edge definitions, including higher-order intralayer or interlayer interactions (e.g., three-body dependencies or multilayer clustering coefficients, multilayer characteristic path length, *etc.*). In what follows, we focus on *true* MLNs—systems that include connections both within and between layers—which provide a natural formalism for hyper-brain or hyper-system dynamics.

This multilayer representation is particularly suited to a synergetic framework of social interaction. It formalizes how *local couplings* within and across subsystems (e.g., neural, autonomic, or behavioral) give rise to emergent *order parameters* that, in turn, constrain and coordinate the components’ activity—an explicit mathematical realization of Haken’s circular causality and enslaving principle. Within this framework, interacting individuals and their subsystems can be represented as nodes in MLNs, where inter-brain, inter-body, and environmental interactions give rise to coupling structures that self-organize over time. The multilayer adjacency tensor or supra-adjacency matrix thus provides a rigorous language for describing and quantifying the self-organization of social systems across hierarchical and physiological scales.

In dyadic or group settings, each modality—such as EEG-derived neural dynamics**,** cardiorespiratory coupling (HRV, respiration), and vocal or motor behavior—constitutes a distinct layer, with nodes representing sensors, brain regions, or effectors. Intralayer edges capture functional connectivity within a given modality (e.g., neural coherence or synchrony), whereas interlayer edges quantify cross-modal couplings (e.g., neural–autonomic, neural–behavioral, or autonomic–behavioral). This allows constructing an empirical *supra-adjacency matrix* that embodies the moment-to-moment configuration of intra- and inter-system interactions across brains and bodies.

**FIGURE 3:**
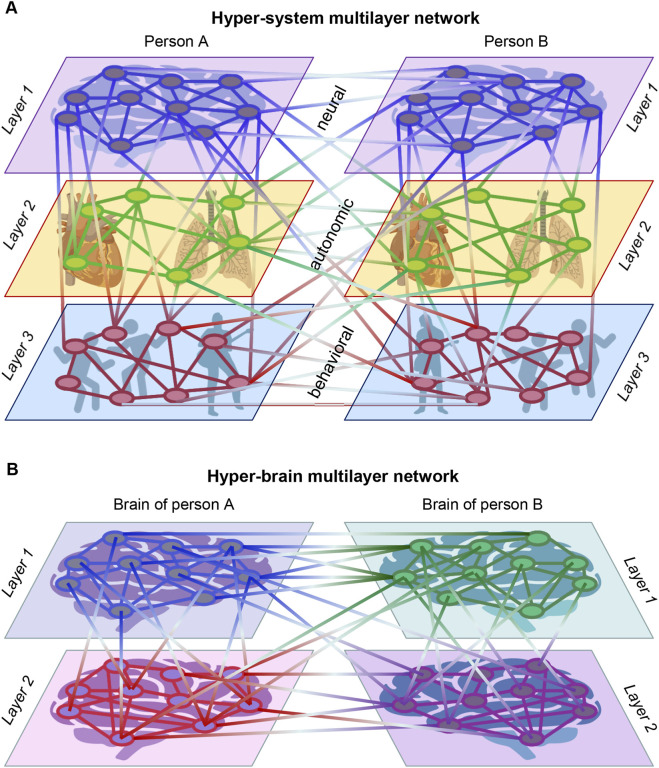
Hyper-system and hyper-brain multilayer networks. **(A)** A three-layer *hyper-system MLN*, comprising neural, autonomic, and behavioral layers for two interacting persons (A and B) Each layer contains a different number of nodes, representing physiological or behavioral variables. Nodes are connected within and between layers, and also within and between individuals, yielding a fully integrated multilayer system. **(B)** A two-layer *hyper-brain MLN*. Nodes represent brain regions or sensors in two interacting brains, connected both within and across layers as well as within and between individuals or brains. In HFNs, intralayer links reflect WFC, interlayer links reflect CFC, and inter-brain couplings can arise through both WFC and CFC. This structure allows quantification of multilayer neural integration within and across brains. Panel B modified from Figure 7C in Müller (2022) under a CC BY 4.0 license.

Figure 3 illustrates this approach by depicting a hyper-system MLN comprising neural, autonomic, and behavioral layers for two interacting individuals (Figure 3A), and a hyper-brain MLN composed of two brains, each represented by two different layers (Figure 3B). As shown, the hyper-system MLN contains layers with different numbers of nodes, reflecting the heterogeneous structure of physiological and behavioral subsystems, whereas the hyper-brain MLN consists of layers with the same number of nodes across brains, reflecting homologous neural architectures. Importantly, both network types are characterized by rich patterns of intra- and interlayer connectivity, which carry functional significance: they encode how processes within and across subsystems become dynamically coordinated during social interaction. Moreover, this multilayer representation offers a principled way to examine how coordination unfolds across temporal scales—for example, by tracking how cross-modal couplings strengthen during periods of increased joint engagement or weaken when the interaction becomes less synchronized. By tracking the evolution of these MLNs over time, one can identify emergent order parameters—for example, collective oscillatory modes, phase alignment, or recurrence patterns—that reflect the self-organized coordination underlying social interaction. Thus, the multilayer operationalization provides a quantitative bridge between theoretical principles of synergetics and measurable indices of interpersonal synchrony.

### Hyper-frequency networks (HFNs) as a specific form of MLNs

In hyperscanning research, measures of oscillatory coupling provide powerful tools for quantifying coordination within and between interacting systems or brains. Two fundamental forms of oscillatory interaction are typically distinguished: within- and cross-frequency coupling (WFC and CFC, respectively). WFC refers to synchronization between neural or other signals oscillating at the same frequency, typically reflecting integration within neural cell assemblies or within-layer interactions of a functional network. By contrast, CFC denotes dependencies between neural/physiological oscillations at different frequencies, which can link local and global processes operating on distinct time scales. This mechanism is thought to facilitate information exchange and coordination between assemblies or layers of organization (Buzsáki and Draguhn, 2004; Buzsáki, 2006; Müller et al., 2016; 2019b; Müller, 2022; Müller and Lindenberger, 2024). The coexistence and interaction of WFC and CFC provide a multidimensional representation of neural dynamics (Jirsa and Müller, 2013). Synchronously oscillating cell assemblies at different frequencies form an efficient substrate for hierarchical integration in the brain, while CFC mechanisms enable phase- or amplitude-specific coordination across these oscillatory domains (Canolty et al., 2006; Klimesch et al., 2008; Doesburg et al., 2009; Canolty and Knight, 2010; Jirsa and Müller, 2013). As Buzsáki (2006) noted, frequency locking can occur between oscillators with integer period ratios, allowing potentially infinite combinations, though constrained by the limited number of coexisting oscillatory classes within the same neuronal substrate. Beyond classical *phase-to-phase* coupling, a wide range of other cross-frequency relationships have been described—including *power-to-power*, *phase-to-power*, *phase-to-frequency*, *amplitude-to-frequency*, and *frequency-to-frequency* coupling (Jensen and Colgin, 2007; De Lange et al., 2008; Jirsa and Müller, 2013; Hyafil et al., 2015; Müller, 2022)—each potentially reflecting distinct pathways of neural integration and information flow. In the work by Lakatos and colleagues (Lakatos et al., 2005), it has been demonstrated that in the primary auditory cortex of awake macaque monkeys, the phase of slow delta oscillations (one to four Hz) modulates the amplitude of faster theta oscillations (4–10 Hz), and in turn, the theta phase modulates the amplitude of gamma oscillations (30–50 Hz). Based on these observations, the authors proposed a hierarchical organization of EEG oscillations, in which the amplitude of higher-frequency activity is systematically governed by the phase of lower-frequency rhythms (Lakatos et al., 2005).

When both WFC and CFC are represented in a single formal structure or a supra-adjacency matrix, the resulting configuration defines a *Hyper-Frequency Network* (HFN). Such networks have been observed within single brains (Müller et al., 2016; 2019b), between interacting brains (Müller and Lindenberger, 2014; 2024), and in complex multi-agent systems such as choir singing (Müller et al., 2018a; 2019a; Delius and Müller, 2023). Within the MLN formalism, each frequency band constitutes a separate layer of nodes (e.g., electrodes or brain regions), where intralayer links encode WFC and interlayer links encode CFC (Brookes et al., 2016; Tewarie et al., 2016; 2021; De Domenico, 2017; Guillon et al., 2017; Buldú and Porter, 2018; Mandke et al., 2018; O’Neill et al., 2018; Tenney et al., 2021). This representation preserves the hierarchical or systemic organization of frequency-dependent processes and allows their simultaneous characterization within a unified topological and dynamical framework.

Conceptually, HFNs exemplify how multilayer modeling can bridge hierarchical self-organization across temporal scales—from rapid local synchronization to slower integrative dynamics spanning multiple agents. In the framework of synergetics, WFC can be viewed as the microscopic coordination among system elements, while CFC mediates mesoscale and macroscale order parameters that constrain or “enslave” the lower-level dynamics. Thus, the HFN formalism provides a mathematically explicit and empirically testable instantiation of circular causality and multi-scale integration in social neurodynamics.

### Dynamical and structural order parameters in MLNs

Within the synergetic framework, *order parameters* capture the macroscopic regularities emerging from the interactions of many microscopic elements. In MLN systems, these order parameters can be broadly divided into *dynamical* and *structural* types. *Dynamical order parameters* describe the collective temporal organization of system components, such as synchronization, phase alignment, or coherence between interacting nodes. In contrast, *structural order parameters* quantify the stable topological configurations that support or constrain these dynamic interactions, such as clustering, path length, betweenness, modularity, *etc.* Together, they represent complementary aspects of self-organization—one reflecting emergent coordination in time, the other the enduring relational architecture that channels such coordination.

At the *local level*, dynamical order parameters capture the coherence among subsets of nodes or within individual layers. For instance, a local order parameter

$$R_{k}e^{i\Psi_{k}\left(t\right)}=\frac{1}{N_{k}}\sum_{j=1}^{N_{k}}e^{i\varphi_{j}\left(t\right)}$$ (2)

measures the degree of phase alignment among *N*_*k*_ nodes within layer *k*, where *R*_*k*_ indicates the local order (ranging from 0 for complete desynchronization to one for perfect synchrony), and *Ψ*_*k*_(*t*) denotes the mean phase of that subnetwork. Analogously, *local structural order parameters* can be expressed by topological measures such as the local clustering coefficient *CC*_*k*_, representing the density of triangular connections within the same layer. Both *R*_*k*_ and *CC*_*k*_ (or other topological measures) thus provide complementary insights into how local coordination emerges dynamically and is supported structurally.

At the *global level*, the overall coherence across the entire MLN can be defined as a weighted aggregation of local order parameters:

$$R_{global}e^{i\eta \left(t\right)}=\frac{1}{N}\sum_{k=1}^{L}{N_{k}R_{k}e}^{i\Psi_{k}\left(t\right)}$$ (3)

where $N=\sum_{k}N_{k}$ represents the total number of nodes across all *L* layers, and *η*(*t*) is the global phase of the system. This measure captures the degree to which different subnetworks (or layers) align their collective states over time. Similarly, a *global structural order parameter* may be defined as the mean or supra-layer clustering coefficient,

$${CC}_{global}=\frac{1}{L}\sum_{k=1}^{L}{CC}_{k}+\frac{1}{L\left(L-1\right)}\sum_{\begin{array}{c} k,l=1\\ k\neq l \end{array}}^{L}{CC}_{kl},$$ (4)

which integrates intra- and interlayer topological organization. Here, *CC*_*k*_ represents the *local order parameters* within individual layers and *CC*_*kℓ*_ denotes the coupling-dependent clustering between layers *k* and *ℓ*. Together, they provide a concise measure of the system’s overall structural coherence—balancing specialization within layers with integration across them.

From this perspective, MLNs provide a natural mathematical substrate for the synergetic description of social and neural systems: *dynamical order parameters* capture temporal coherence and coordination (e.g., inter-brain synchronization, rhythmic entrainment), whereas *structural order parameters* reflect the enduring relational topology that stabilizes and channels these interactions. The interplay between them—structure guiding dynamics and dynamics reshaping structure—embodies the core of circular causality in self-organizing social systems.

It should also be noted that the local order parameters described above for specific layers can be extended to the level of individual nodes, both within and across layers. In this formulation, each node’s contribution to the local order reflects its phase alignment or structural embedding relative to its neighbors, thus representing a micro-level indicator of local coherence or network topology. Such node-specific order parameters become particularly informative in hyperscanning paradigms, where nodes correspond to different signals or frequencies of interacting individuals. Tracking how these local parameters evolve within and between layers allows one to capture how individual contributions shape, and are constrained by, the emergent global organization of the multilayer system.

## The synergetic multilayer social interaction hypothesis (SMSIH)

A growing body of research in cognitive neuroscience, psychophysiology, and nonlinear dynamics suggests that social interaction is not merely the sum of two or more isolated information-processing systems. Rather, interacting individuals form coupled dynamical systems whose joint behavior is governed by emergent collective variables (Kelso, 1995; Oullier et al., 2008; Sänger et al., 2011; Tognoli and Kelso, 2015; Hasson and Frith, 2016; Tognoli et al., 2018; 2020; Müller et al., 2021; Müller, 2022; Doering et al., 2024). Synergetics (Haken, 1983; 1984; 1997; 2006; 2016) provides a formal framework for understanding how such collective patterns arise from the reciprocal influences among components. Central to this framework is the concept of circular causality, whereby local interactions among system elements give rise to global, low-dimensional order parameters, which in turn constrain and “enslave” the microdynamics of the elements (Haken, 1983; Jirsa et al., 1994; Jirsa and Haken, 1996).

Based on this view, we propose the *SMSIH*, which states that social interaction emerges and stabilizes through self-organizing processes in MLNs, where circular causation between neural, physiological, and behavioral levels gives rise to synergetic patterns of interpersonal coordination. In this framework, order parameters play a central role in guiding system evolution and enabling transitions between coordination regimes. Social interaction thus becomes a process of *shared self-organization*, in which information flows recurrently across subsystems within and between individuals. When this circular causation is sufficiently strong or exceeds a critical threshold, the dyad or group converges on shared *order parameters*—low-dimensional collective variables that compress the high dimensionality of neural, autonomic, and behavioral activity into a smaller set of patterns governing joint behavior.

These order parameters may be *dynamical,* quantify emergent temporal patterns in the coupled system (e.g., joint oscillatory modes, shared phase relations, collective entropy levels), or *structural,* capturing stable or metastable network configurations (e.g., global topology measures, stable cross-brain or cross-system network modules, or multilayer motifs). Both types of order parameters provide empirically testable signatures of emergent interpersonal organization. The hypothesis therefore predicts that stronger and more symmetrical coupling across neural, autonomic, and behavioral layers should yield more robust and persistent alignment between interacting individuals, as reflected in the stabilization and persistence of such macro-level order parameters over time.

Within this hypothesis, *stronger and more symmetrical cross-layer couplings*—for instance, between neural and autonomic oscillations, between respiratory and vocal dynamics, or between corresponding EEG frequency bands across two or more brains—are predicted to lead to.

- More stable interpersonal coordination,
- Faster convergence on shared patterns,
- Greater resistance to perturbations,
- Tighter alignment of neural, physiological, and behavioral systems or signals.

While symmetric coupling may enhance alignment robustness in dyadic or egalitarian interactions, the SMSIH framework also accommodates functional asymmetry, where directional influences from designated leaders or conductors stabilize coordination in hierarchical contexts. Symmetry is not a requirement for self-organization, but rather one of several regimes in which emergent order parameters can stabilize collective dynamics.

To make the framework explicitly testable, the SMSIH generates several empirically falsifiable predictions concerning the emergence, stability, and multilayer organization of social coordination (see Table 1). In self-organizing coordination systems, stability typically occurs within an intermediate range of coupling strengths. While weak coupling fails to produce collective order, excessively strong coupling may destabilize coordinated states by amplifying perturbations, suppressing dynamical flexibility, or imposing incompatible constraints across interacting layers. Such effects are well known in coordination dynamics and nonlinear oscillator theory, where strong coupling can induce phase transitions, metastable switching, or oscillation quenching.

**TABLE 1:** Testable predictions of the synergetic multilayer social interaction hypothesis (SMSIH).

Predictions
Prediction 1: Emergence of collective order parameters As interpersonal coupling increases, coordinated social systems should exhibit the emergence of macroscopic order parameters that constrain the dynamics of individual components. Empirically, this should manifest as increased synchronization across individuals in neural, physiological, or behavioral signals
Prediction 2: Nonlinear phase transitions in coordination dynamics Social coordination should exhibit nonlinear transitions from incoherent to coordinated states when interpersonal coupling exceeds critical thresholds. These transitions should be observable as abrupt increases in inter-brain or inter-behavioral coupling measures
Prediction 3: Multilayer amplification of synchronization Coordination should be stronger and more stable when coupling occurs simultaneously across multiple layers (e.g., neural, physiological, behavioral) compared to single-layer coupling. Multilayer network analysis should reveal increased cross-layer coupling and enhanced global network coherence
Prediction 4: Metastability of social coordination Rather than maintaining permanent synchronization, interacting individuals should exhibit metastable dynamics characterized by intermittent synchronization and desynchronization. Such dynamics should appear as temporal fluctuations in coupling strength and switching between coordination patterns
Prediction 5: Instability under excessive coupling Beyond an optimal range, excessively strong coupling may destabilize coordinated states. Empirically, this should manifest as phase slips, loss of coordination stability, or transitions between competing coordination patterns
Prediction 6: Circular causation across scales Macroscopic coordination patterns should exert top-down constraints on individual dynamics while simultaneously emerging from bottom-up interactions. This circular causation should be observable as bidirectional influences between system-level order parameters and local interaction dynamics

Importantly, the hypothesis integrates insights across multiple domains: neuroscience, physiology, and behavior, revealing the alignment of movement, timing, and expressive features in joint action. From this perspective, interpersonal coordination arises when these multiple layers jointly self-organize around one or more order parameters that stabilize the relational dynamics between individuals. Such a multilevel approach allows the formulation of precise, testable predictions about how social synchrony emerges and changes over time, and how disruptions at one layer propagate through the multilayer system. In the following sections, we present several examples of MLNs from physiology and cognitive neuroscience to illustrate how social interaction can be conceptualized as a multilayer phenomenon from a synergetic perspective.

### Social interaction as a multilayer network phenomenon

As noted by Buzsáki (2010), cognitive neuroscience increasingly frames biological systems as integrated networks, in which subsystems interact continuously across feedback loops and multiple spatio-temporal scales. These interactions optimize collective function and give rise to dynamic, state-dependent patterns of coordination (Bartsch et al., 2015; Ivanov, 2021). From a network perspective, physiological states are reflected in both the topology and the strength of couplings between subsystems, illustrating the tight interplay between structure and function (Bashan et al., 2012; Faes et al., 2014; Bartsch et al., 2015; Liu et al., 2015; Ivanov et al., 2016; Rizzo et al., 2020; Ivanov, 2021).

Using time delay stability (TDS) measures, Ivanov and colleagues showed that transitions between sleep stages involve systematic reconfigurations of network connectivity across multiple physiological systems—including cardiac and respiratory activity, muscle tone, eye and leg movements, and spectral brain activity across distinct EEG frequency bands (Bashan et al., 2012; Ivanov et al., 2016). For instance, deep sleep was characterized primarily by brain–brain connectivity, while lighter sleep stages and wakefulness involved increasing interactions between brain and peripheral systems. Cortico-muscular coupling, especially within the same frequency band (WFC), was strongest during wakefulness and REM sleep, and weaker in deep sleep (Rizzo et al., 2020). Beta-band EEG activity acted as a hub in the brain–heart network, mediating bidirectional information flow between cortical and peripheral systems (Faes et al., 2014).

Recent studies within the Network Physiology framework demonstrate that cortico–muscular and cardio–muscular interactions form structured, state-dependent networks that reorganize with physiological demands, fatigue, and pathology. Cardio–muscular coupling during exercise exhibits frequency-specific interaction profiles reflecting muscle function and fiber composition, with coupling strength systematically declining and shifting from synchronous to asynchronous dynamics under fatigue (Garcia-Retortillo et al., 2025). Similarly, inter-muscular coordination during maximal exercise is organized into hierarchical, modular networks that reorganize with fatigue along distinct phase-space trajectories, pointing to general principles of motor coordination (Garcia-Retortillo et al., 2023).

Extending this approach to brain–muscle interactions, network analyses across wakefulness and sleep reveal state-specific hierarchical cortico–muscular networks, with distinct brain rhythms acting as dominant mediators of muscle control (Rizzo et al., 2022; 2023). In healthy individuals, coupling strength is stratified across physiological states, whereas Parkinson’s disease is associated with a breakdown and reorganization of cortico–muscular connectivity, disrupting synchronous communication and state-dependent network structure (Rizzo et al., 2023). Together, these studies reveal previously unrecognized principles of multilevel physiological coordination and highlight the potential of network-based markers for characterizing fatigue, fitness, physiological states, and neurodegenerative disorders.

These findings provide a natural foundation for conceptualizing hyper-brain or hyper-system interactions using an MLN approach. By formally treating multiple coupled subsystems as a multilayer structure, one can quantify both local and global order parameters, capture emergent circular causation, and describe how individual components collectively give rise to coherent, system-wide dynamics. This perspective directly motivates the construction of MLNs in dyadic and group contexts—such as romantic kissing (Müller and Lindenberger, 2014), musical ensemble performance (Müller, 2022; Müller and Lindenberger, 2024), and choral singing (Müller et al., 2018a; 2019a; 2026; Delius and Müller, 2023)—where intra- and inter-brain couplings, or cross-subsystem interactions, across multiple frequency layers provide the structural backbone for an integrated hyper-system representation.

At present, MLN approaches have not yet been systematically applied to the study of social synchrony. The studies cited here therefore primarily serve to illustrate how such a framework can be operationalized empirically. At the same time, a growing body of research from independent laboratories has documented interpersonal synchronization across neural, physiological, and behavioral domains, providing a broad empirical basis for the multilayer perspective proposed here (Keller et al., 2014; Czeszumski et al., 2020; Hamilton, 2021; Moreau and Dumas, 2021; Lange et al., 2022; Sadaphal et al., 2025; Curzel et al., 2026).

#### Hyper-system hyper-frequency MLNs during choral singing

**FIGURE 4:**
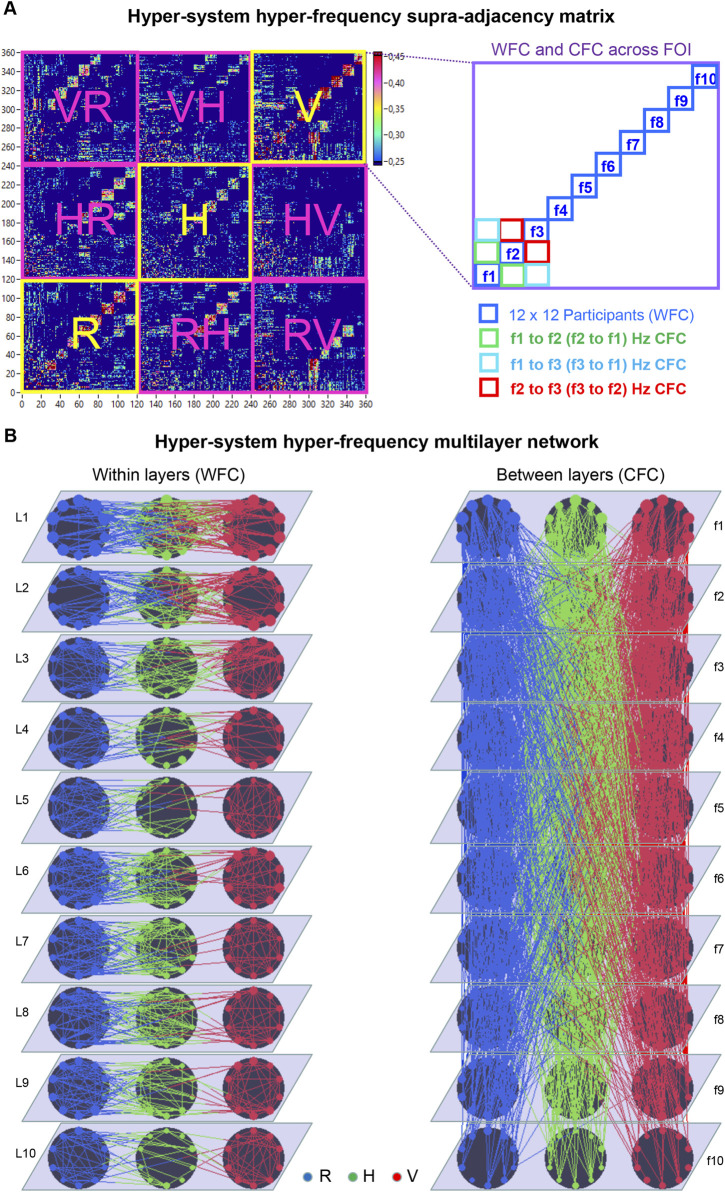
Hyper-system hyper-frequency supra-adjacency matrix and corresponding MLN during choral singing. **(A)** Hyper-system hyper-frequency supra-adjacency matrix. The matrix on the left comprises 360 nodes resulting from the combination of 12 choir members, 10 frequencies, and three subsystems. The structure of the matrix, including the ordering of choir members and frequencies as well as the representation of WFC and CFC, is shown on the right. **(B)** Hyper-system hyper-frequency MLN. This MLN consists of 10 layers corresponding to the 10 frequencies, with three subsystems depicted by black circles and 12 choir members arranged within each circle. The left panel represents WFC intralayer connections within and between the three subsystems, indicated by different colors. The right panels represent CFC interlayer connections within and between the three subsystems. WFC = within-frequency coupling; CFC = cross-frequency coupling; R = respiration; H = HRV; V = voice; FOI(Layers): f1 (L1) = 0.025; f2 (L2) = 0.050; f3 (L3) = 0.075; f4 (L4) = 0.100; f5 (L5) = 0.125; f6 (L6) = 0.150; f7 (L7) = 0.200; f8 (L8) = 0.250; f9 (L9) = 0.300; f10 (L10) = 0.400 Hz.

To illustrate the concept of social MLNs in physiological coordination, we refer to a choir study involving 11 singers and a conductor, where WFC and CFC among respiratory, cardiac, and vocal subsystems together with the conductor’s hand movements, oscillating at ten distinct frequencies, were used to construct a HFN or MLN, shown in Figure 4A as a hyper-system adjacency matrix (*cf.* Müller et al., 2018a; 2019a). Figure 4B depicts the MLN, where each layer represents one of the ten frequency components. WFC defines intra-layer connections between choir members within and across the three subsystems, while CFC mediates inter-layer communication. For simplicity, the conductor’s hand movements were omitted in this representation.

Interestingly, coupling strengths within the choir were higher when singing a canon in unison than when performing it in parts (*cf.* Müller and Lindenberger, 2011), but this effect was limited to WFC, that is, within-layer connections. In contrast, CFC—or connectivity between the layers—was particularly enhanced during canon singing in parts, supporting the coordination of temporally shifted melodic entries and suggesting that intra- and interlayer connections subserve distinct functional roles. Moreover, WFC between choir members was stronger at higher frequencies (0.15–0.40 Hz), particularly within subsystems, whereas CFC dominated at lower frequencies (0.025–0.05 Hz), both within and between subsystems, suggesting that faster oscillations contribute to fine-grained interpersonal alignment, while slower oscillations orchestrate large-scale integration of the ensemble (Müller et al., 2018a).

Network modularity analyses revealed that the number of *hub nodes* (nodes with strong intra-module connectivity) and, in particular, *connector nodes* (linking distinct modules or communities) increased when the canon was sung in parts, whereas the number of modules remained constant across conditions. This pattern suggests that polyphonic coordination requires a richer intermodular architecture and thus engages more nodes serving integrative or controlling functions (Müller et al., 2018a). From a synergetic viewpoint, these findings imply that both *local order parameters* (e.g., frequency-specific WFC) and *global order parameters* (e.g., CFC linking across modules and frequencies) are essential for maintaining coherence in distributed, multilayer coordination systems and appear to fulfill complementary functional roles. The transition from unison to canon singing can be viewed as a change in control parameters—such as temporal phase shifts and melodic differentiation—that drive the system from a highly ordered collective state toward a more complex, self-organized regime in which global coherence emerges from the interplay of multiple, partially independent subsystems.

Follow-up analyses (Müller et al., 2019a) demonstrated further differences in HFN or MLN topology between singing conditions. When performing the canon in parts, the clustering coefficient and local and global efficiency were higher, and the characteristic path length shorter, than during unison singing. These results indicate a small-world network (SWN) organization—characterized by high local clustering and short global distances—optimizing both segregation and integration of information flow within the HFN, particularly, when singing the canon in parts. Such SWN properties are recognized as a universal hallmark of biological and social systems that must balance local specialization with global coordination (Müller et al., 2018a; 2019a; 2021; Müller, 2022; Delius and Müller, 2023). Within the synergetic MLN framework, these measures represent structural order parameters, with *local metrics* (clustering coefficient, local efficiency) reflecting the emergence of coherent subsystems, and *global metrics* (characteristic path length, global efficiency) indexing system-wide integration.

From this perspective, choir singing can be understood as a paradigmatic case of social self-organization, in which dynamic (phase-based) and structural (topological) order parameters jointly govern the emergence of coordinated collective behavior. Future analyses could extend this approach by examining whether *node-level* structural order parameters—such as local clustering within and between frequency layers—also contribute to multilayer coordination across interacting physiological systems.

##### Multilayer recurrence networks during singing

In recent work (Müller et al., 2026), recurrence quantification analysis (RQA) was employed to capture the nonlinear dynamics underlying choral singing by combining recurrence plot (RP) and joint recurrence plot (JRP) components to construct a multilayer recurrence network (MLRN). This multilayer framework enables the characterization of multi-level coordination dynamics essential for synchronized group performance, and provides a natural perspective for synergetic analysis (Müller et al., 2021; Müller, 2022). In this approach, interactions within the same subsystem across different individuals define distinct network layers, whereas interactions between different subsystems represent cross-domain coupling, reflecting the integration of diverse physiological processes across these layers. Such a framework is consistent with prior approaches in which each component of a system is modeled as a separate recurrence network layer within a multiplex or multilayer network (Feldhoff et al., 2012; Eroglu et al., 2018; Zou et al., 2019; Hasselman and Bosman, 2020; Marwan and Kraemer, 2023), and it aligns with the HFN perspective described above.

RQA is based on the reconstruction of the system’s phase space and the examination of its dynamical states, resulting in a corresponding *N* × *N* recurrence matrix defined as:

$$R_{ij}=\left\{\begin{array}{c} 1:x_{i}\approx x_{j}\\ 0:x_{i}≉x_{j} \end{array}\right.,i,j=1,\ldots ,N$$ (5)

where *N* is the number of states considered, and $x_{i}\approx x_{j}$ denotes approximate equality up to a threshold *ε*. This threshold is critical, since real-world systems rarely return exactly to a previous state but only approximately. Formally, the recurrence matrix can be expressed as (Marwan et al., 2007):

$$R_{ij}\left(\varepsilon \right)=\Theta \left(\varepsilon -\left\|x_{i}-x_{j}\right\|\right),i,j=1,\ldots ,N$$ (6)

where *Θ*(⋅) is the Heaviside function, and bracket ‖∙‖ denotes a norm (here, the Euclidean norm). In this study (Müller et al., 2026), a fixed recurrence rate of 10% was used for all RPs, with the threshold *ε* adjusted accordingly for each subsystem and choir member.

To study interactions between different individuals within and between subsystems, a *joint recurrence plot* (JRP) approach was applied, capturing the simultaneous recurrences of two choir members in their respective phase spaces. For two signals *x* and *y*, the joint recurrence matrix is defined as (Marwan et al., 2007):

$${JR}_{i,j}^{x,y}\left(\varepsilon^{x},\varepsilon^{y}\right)=\Theta \left(\varepsilon^{x}-\left\|x_{i}-x_{j}\right\|\right)\cdot \Theta \left(\varepsilon^{y}-\left\|y_{i}-y_{j}\right\|\right),i,j=1,\ldots ,N.$$ (7)

A joint recurrence occurs when *x*_*j*_ returns near a former state *x*_*i*_, simultaneously with *y*_*j*_ returning near *y*_*i*_, thus reflecting the joint probability of coordinated dynamical recurrences (Marwan et al., 2007).

Moreover, using RPs and the corresponding JRPs, the directionality of coupling between two systems can be inferred from the conditional probabilities of recurrence. The mean conditional probabilities of recurrence (*MCR*) between systems *X* and *Y* are defined as follows (Romano et al., 2007):

$$MCR\left(Y|X\right)=\frac{1}{N}\sum_{i=1}^{N}\left({\vec{y}}_{i}|{\vec{x}}_{i}\right)=\frac{1}{N}\sum_{i=1}^{N}\frac{\sum_{j=1}^{N}{JR}_{i,j}^{X,Y}}{\sum_{j=1}^{N}R_{i,j}^{X}},$$ (8)

and

$$MCR\left(X|Y\right)=\frac{1}{N}\sum_{i=1}^{N}\left({\vec{x}}_{i}|{\vec{y}}_{i}\right)=\frac{1}{N}\sum_{i=1}^{N}\frac{\sum_{j=1}^{N}{JR}_{i,j}^{X,Y}}{\sum_{j=1}^{N}R_{i,j}^{Y}},$$ (9)

where *p* (*y*_*i*_|*x*_*i*_) estimates the probability that the trajectory of *Y* recurs to the neighborhood of *y*_*i*_ under the condition that the trajectory of *X* recurs to the neighborhood of *x*_*i*_. Conditional probability *p* (*x*_*i*_|*y*_*i*_) is defined analogously. Directionality can then be inferred by comparing these two conditional probabilities: If *X* drives *Y*, then *MCR* (*Y*|*X*) is less than *MCR* (*X*|*Y*), and if *Y* drives *X*, the opposite holds. Directional coupling thus provides an important supplement for characterizing dynamical interactions both within and between subsystems.

**FIGURE 5:**
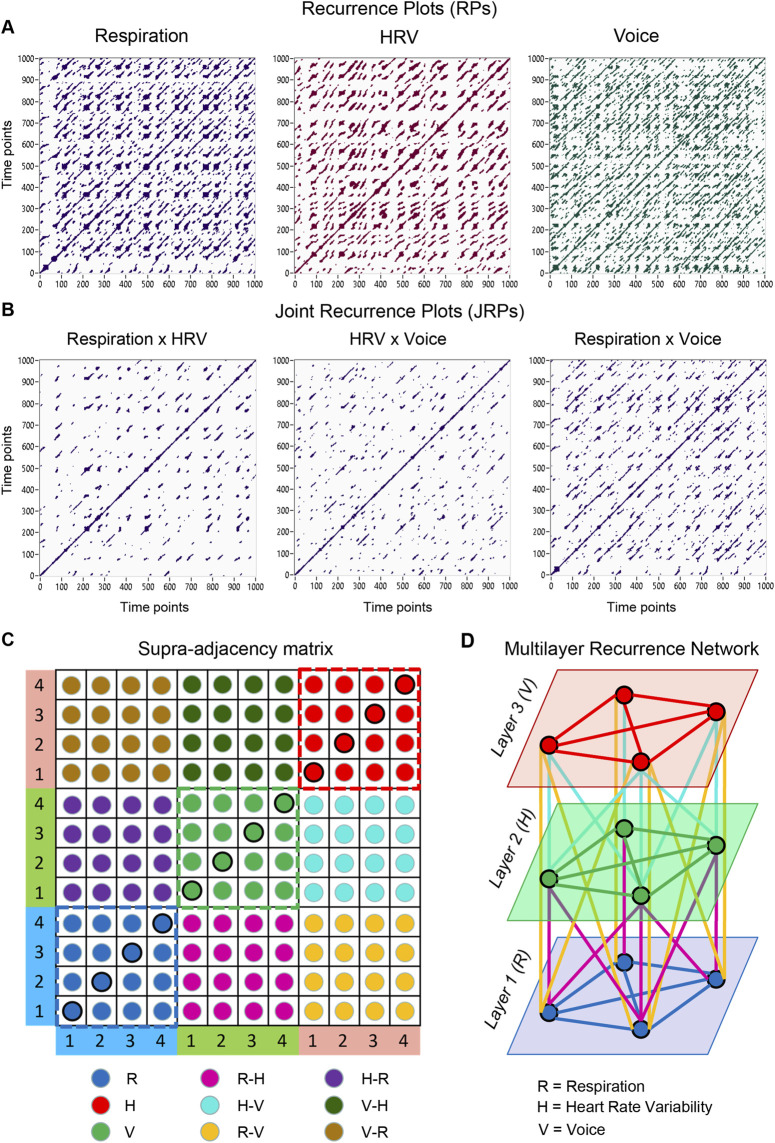
Recurrence plots (RPs), joint recurrence plots (JRPs), supra-adjacency matrix, and corresponding multilayer recurrence network (MLRN). **(A)** RPs illustrating the internal dynamics of each subsystem during singing. **(B)** JRPs depicting the interactions between the subsystems shown in **(A)**. Data shown in **(A)** and **(B)** are from a single participant. **(C)** Schematic illustration of a supra-adjacency matrix constructed for four singers and three subsystems. Circles correspond to specific RP or JRP measures, with colors denoting subsystems and their interactions. Circles along the main diagonal correspond to individual RPs. Intralayer connections appear in diagonal blocks (indicated by dashed squares), whereas interlayer connections occupy the off-diagonal blocks, reflecting couplings between layers. **(D)** Multilayer recurrence network (MLRN) based on the bottom-right portion of **(C)**. In this network, edges within layers represent connections between choir members within individual subsystems, while edges between layers indicate the interactions between subsystems, both within and between choir members. Note that edges in the MLRN correspond JRP indices. Subsystems: R, respiration; H, HRV; V, Voice. Subsystem interactions: R-H, H-V, R-V, H-R, V-H, and V-R. The full matrix (bottom-right and top-left off-diagonal portions) illustrates a directed network derived from mean conditional probabilities of recurrence (*MCR*). Panels A and B modified from Figures 2B,C, and panels C and D modified from Figures 3A,B in Müller et al. (2026), under a CC BY 4.0 license.

Figures 5A,B present individual RPs for the three subsystems under investigation (respiration, HRV, and voice) together with their corresponding JRPs. The MLRN is constructed from a supra-adjacency matrix in which RP indices along the main diagonal represent subsystem-specific dynamics of individual choir members, while off-diagonal JRP indices represent within- and between-member couplings both within and across subsystems. For illustration, Figure 5C provides a schematic example of such a supra-adjacency matrix for four choir members across three subsystems. Each circle corresponds to an RP or JRP measure, with color coding indicating the relevant subsystem or subsystem pair. Because this supra-adjacency matrix is largely symmetric (except for *MCR*-based directional coupling), the lower-right block is typically sufficient for constructing the network; for directional coupling analyses, the full matrix is required. This supra-adjacency matrix forms the basis of the MLRN schematically depicted in Figure 5D. In this network, intralayer edges reflect the dynamical behavior of choir members within each physiological subsystem, whereas interlayer edges capture cross-subsystem interactions both within and across individuals.

The MLRN framework provides a powerful tool for examining temporal transitions, intermittencies, and regime shifts in complex systems, offering deep insights into real-world social coordination processes such as choral singing (Feldhoff et al., 2012; Eroglu et al., 2018; Zou et al., 2019; Hasselman and Bosman, 2020; Marwan and Kraemer, 2023; Müller et al., 2026). By applying RQA across multiple physiological subsystems, the MLRN captures small-scale recurrence structures—specifically, recurrence point density as well as diagonal and vertical line configurations—reflecting deterministic and laminar aspects of subsystem dynamics, respectively. Interpreting recurrence or joint recurrence matrices as adjacency matrices enables the derivation of graph-theoretical metrics that quantify higher-order properties of the system, thereby bridging dynamical systems analysis with network theory (Müller et al., 2026). Importantly, different RQA measures can be used to populate the supra-adjacency matrix, each contributing complementary information about the evolving network dynamics and together providing a more complete characterization of the system.

Importantly, RQA is particularly well-suited for complex, nonlinear, and non-stationary systems (Hatipoğlu and Yılmaz, 2026). Recurrence plots and derived metrics (e.g., determinism, laminarity) can detect structural changes, trends, and transitions without being biased by non-stationarity. Similarly, time-windowed linear coupling measures, as used in the cited studies (see below), mitigate the impact of non-stationarity by computing functional connections over short overlapping intervals. Consequently, although social interactions naturally exhibit non-stationary dynamics, these methodological safeguards ensure that the recurrence structures and network couplings identified reflect true metastable coordination rather than transient coincidences (Müller et al., 2026).

Using MLRN approach, distinct dynamic signatures were observed for the respiratory, cardiac (HRV), and vocal subsystems. HRV exhibited the highest determinism, laminarity, and complexity—particularly during rest—suggesting a stable yet richly structured dynamic regime. In contrast, the respiratory subsystem became dominant during active singing, exerting strong influence on the coordination between subsystems. Singing markedly altered the recurrence dynamics, leading to greater synchronization and higher-dimensional integration within and across participants compared to rest. Furthermore, the specific mode of ensemble singing modulated these dynamics: singing in unison was characterized by more fragmented and less integrated patterns within the voice subsystem, whereas singing in parts fostered cross-subsystem coupling and dynamic integration across multiple temporal and physiological layers (Müller et al., 2026).

From a *synergetic* standpoint, these findings highlight how local and global order parameters—emerging from the interplay of physiological subsystems within and between choir members—govern the self-organization of the ensemble. The MLRN framework thus not only captures transient coordination and reorganization processes but also reveals how collective patterns evolve through mutual adjustment of subsystems at different temporal scales and recurrence dynamics. In this sense, the MLRN acts as a bridge between microscopic (individual-level) dynamics and macroscopic (ensemble-level) order formation, providing a quantitative lens on how shared rhythmic structures and physiological couplings give rise to emergent, self-organized states of social synchrony (Müller et al., 2021; 2026; Müller, 2022).

### Intra- and inter-brain synchronization and hyper-brain networks

A growing body of research using hyperscanning methods has revealed that social interaction and joint action—such as making music, dancing, or engaging in cooperative or competitive tasks—depend critically on both intra- and inter-brain synchronization (Lindenberger et al., 2009; Astolfi et al., 2010; 2020; Dumas et al., 2010; 2012; 2020; Sänger et al., 2012; 2013; Müller et al., 2013; 2018b; Keller et al., 2014; Acquadro et al., 2016; Müller and Lindenberger, 2019; 2022; 2023; 2024; Czeszumski et al., 2020; Gugnowska et al., 2022). Together, these two forms of synchronization give rise to *hyper-brain network* (HBN) activity—a higher-order neural dynamic reflecting the integrated state of interacting agents (Sänger et al., 2012; Müller et al., 2013; 2018b; 2021; Müller and Lindenberger, 2019; 2022; 2023; 2024; Müller, 2022). This HBN activity typically increases during phases of intense social coordination and exhibits both temporal and structural reorganization in response to changing situational demands (Sänger et al., 2011; 2012; 2013; Dumas et al., 2012; Yun et al., 2012; Müller et al., 2013; 2018b; 2021; Hu et al., 2017; Szymanski et al., 2017b; Ahn et al., 2018; Müller and Lindenberger, 2019; 2022; 2023; 2024; Stone et al., 2019; Astolfi et al., 2020; Balconi et al., 2020; Sadaphal et al., 2025).

An important conceptual distinction should be emphasized here: while intra-brain synchronization is mostly substrate-bound (mediated by anatomical and functional connections within one brain), inter-brain synchronization is substrate-free—there are no direct physical connections between brains. Instead, synchronization across brains likely relies on shared temporal structures emerging from joint perception, coordinated motor action, and mutual prediction (Lindenberger et al., 2009; Sänger et al., 2012; Müller and Lindenberger, 2019). Notably, several studies have shown that inter-brain synchrony cannot be fully reduced to shared input or output but displays intrinsic self-organizing tendencies, reflecting mutual entrainment and collective timing (Lindenberger et al., 2009; Sänger et al., 2012; 2013; Szymanski et al., 2017b; Gvirts and Perlmutter, 2020; Müller et al., 2021; Novembre and Iannetti, 2021; Reinero et al., 2021; Gugnowska et al., 2022; Lender et al., 2023).

Despite substantial progress, the causal relations between intra- and inter-brain dynamics remain incompletely understood (Kingsbury and Hong, 2020; Müller, 2022). Recent evidence suggests that *within-brain connectivity* reflects an individual’s current internal state, while *between-brain connectivity* facilitates the adjustment and alignment of these states during interpersonal interaction (Müller et al., 2021; Shamay-Tsoory, 2021; Müller, 2022). Thus, social coordination can be conceptualized as a continuous circular process, in which cell assemblies within each brain (local order parameters) are dynamically coupled and re-aligned through between-brain synchrony (global order parameters). Extending Hebb’s original notion of cell assemblies, a *hyper-brain cell assembly hypothesis* has been suggested (Müller, 2022). This framework suggests that coupled neural assemblies can transiently emerge across different brains through inter-brain synchrony, effectively rephrasing Hebb’s rule—“what fires together, wires together”—in a functional rather than anatomical sense. Within this perspective, “wiring” refers to dynamically established functional connectivity rather than permanent synaptic links. Importantly, the coupling between brains is mediated through shared sensorimotor channels—such as visual perception, auditory signals, and coordinated movement—which allow neural activity in one individual to influence neural dynamics in another. Thus, inter-brain synchronization does not imply direct anatomical connections but arises from reciprocal interactions mediated by the environment (*cf.* Müller and Lindenberger, 2019). Hyper-brain cell assemblies thus constitute transient yet functionally integrated units spanning two or more brains, which jointly ignite during episodes of synchronized social behavior (Müller, 2022).

From a synergetic perspective, a *hyper-brain cell assembly* can be conceptualized as a superordinate system governed at the macro level by one or more *order parameters*. These order parameters define the collective state of the system and thereby “enslave” the behavior of its constituent neuronal elements across the interacting brains. At the same time, the micro-level dynamics of these neuronal elements (e.g., neurons or synapses) feed back to influence the structure and temporal evolution of the hyper-brain cell assembly as a whole—an expression of the principle of *circular causality* (Haken, 2006; 2016). Crucially, this recursive interplay of top-down and bottom-up processes operates not only within individual brains but also across brains that are dynamically coupled through synchronization, forming a hyper-brain network or assembly that functions as a superordinate system or superorganism (Müller et al., 2021; Müller, 2022). Through repeated joint activity, unnecessary degrees of freedom are gradually reduced, leading to the emergence of stable coordination patterns—a hallmark of social self-organization.

Empirical evidence supports this view. In studies with guitar duets and quartets, modules comprising nodes from two or more brains—termed *hyper-brain modules*—were identified as strongly interconnected communities across brains that may represent prototypes of such hyper-brain cell assemblies (Sänger et al., 2012; Müller et al., 2013; Müller and Lindenberger, 2014; 2019; 2023; Müller, 2022). These modules dynamically reconfigure depending on task structure and interaction demands, reflecting the adaptive integration of intra- and inter-brain dynamics and corresponding hyper-brain networks (Müller et al., 2021; Müller, 2022). Comparable patterns of hyper-brain modular organization have also been observed in hyper-frequency (or multilayer) networks during intimate and cooperative behaviors such as kissing (Müller and Lindenberger, 2014) and in multi-system HFNs during singing (Müller et al., 2018a), suggesting that hyper-brain or hyper-system modularity represents a general organizational principle underlying coordinated social interaction. Similar concepts have been articulated by (Shamay-Tsoory, 2021), who proposed *inter-brain plasticity*—the capacity of inter-brain networks to reorganize their structure through repeated interaction-based learning (Shamay-Tsoory, 2021).

Converging evidence from animal studies also supports the existence of cell-level inter-brain coupling. In a hyperscanning study with mice, it has been shown that inter-brain correlations arise from cell assemblies encoding self- and other-related behaviors, with dominant individuals exerting stronger influence on synchrony patterns (Kingsbury et al., 2019). Similarly (Zhang and Yartsev, 2019), demonstrated that correlated spiking activity between bats covaried with the degree of social interaction. These findings suggest that correlated neural activity across brains—at the scale of local assemblies—constitutes the microscopic substrate for macro-level hyper-brain synchronization, consistent with the proposed hyper-brain cell assembly framework.

From a multilayer perspective, intra- and inter-brain links can be understood as distinct but interacting layers or as different aspects within a social MLN (*cf.* Kivelä et al., 2014; De Domenico, 2022). Local and global order parameters can then be derived from these structures to quantify emergent coordination patterns at both individual and collective levels—a central step toward integrating synergetics, network neuroscience, and social interaction research.

#### Hyper-brain MLNs during kissing

**FIGURE 6:**
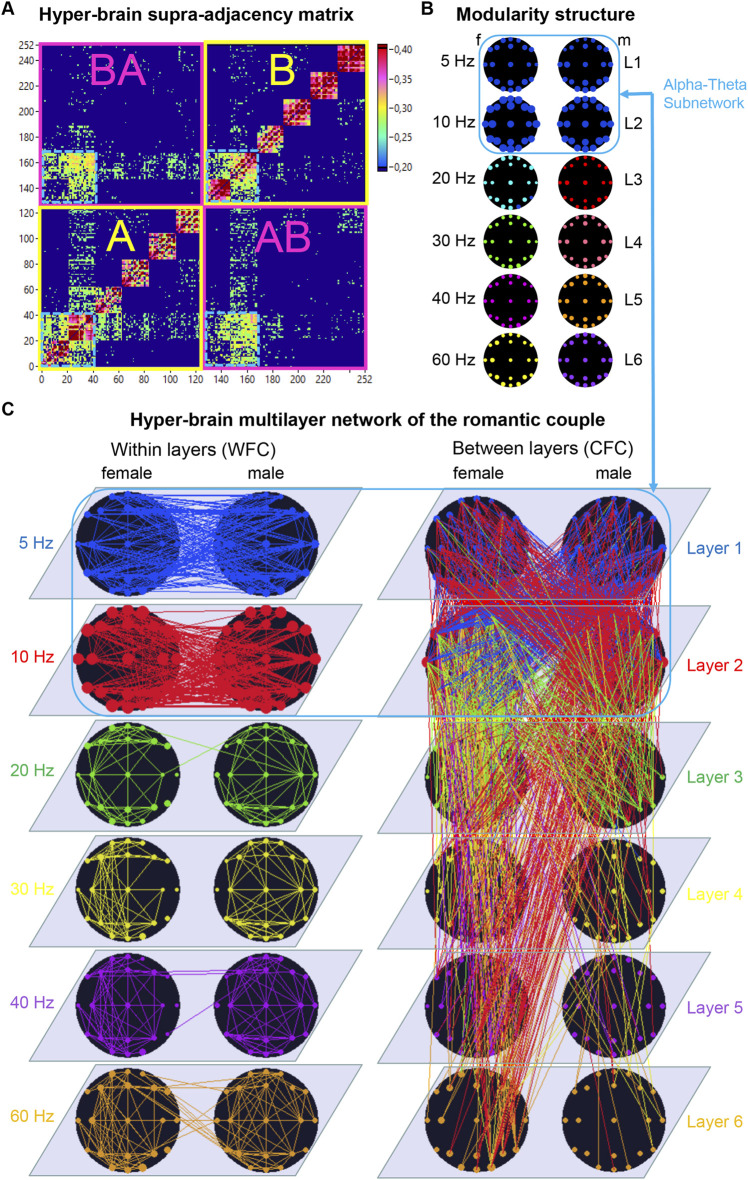
Hyper-brain supra-adjacency matrix, modularity structure, and corresponding MLN of the couple during romantic kissing. **(A)** Hyper-brain supra-adjacency matrix. The matrix comprises 252 nodes resulting from the combination of 21 electrode sites and six oscillation frequencies within and between the female **(A)** and male **(B)** partners’ brains. **(B)** Modularity structure of the full MLN. Modularity analysis identified nine distinct modules, indicated here by different colors. The largest module—the so-called theta–alpha module—consists of 5- and 10-Hz oscillatory nodes (indicated in blue) in both partners’ brains. Blue dashed rectangles mark this theta–alpha module in the supra-adjacency matrix. The remaining modules represent frequency-specific subnetworks that are largely segregated across the two brains. **(C)** A six-layer hyper-brain MLN of the romantic couple. This MLN comprises six layers corresponding to the six oscillation frequencies, indicated by different colors, for both partners. The left panel shows WFC intralayer connections within and between the two brains; the right panel shows CFC interlayer connections, also within and between brains. WFC = within-frequency coupling; CFC = cross-frequency coupling (*cf.* Müller and Lindenberger, 2014; Müller et al., 2021; Müller, 2022).

To illustrate the construction and interpretation of hyper-brain multilayer networks (HB-MLNs) in social interaction, we refer to a study investigating romantic couples during kissing (Müller and Lindenberger, 2014). In this work, WFC and CFC were computed across 21 EEG channels per brain and six distinct oscillation frequencies to construct a HB-MLN comprising both intra- and inter-brain connections (see hyper-brain supra-adjacency matrix in Figure 6A).

Network analyses revealed that hyper-brain connectivity during *partner-oriented kissing*, compared to *hand kissing*, was characterized by increased connection strength and reduced characteristic path length, indicating enhanced integration and optimized information transfer both within and between the partners’ brains. The simultaneous strengthening of intra- and inter-brain coupling suggests that neural assemblies synchronize not only locally within each brain but also across brains—thereby giving rise to *hyper-brain cell assemblies*. This enhanced inter-brain synchrony underscores the evolutionarily adaptive role of affiliative behaviors such as kissing in fostering interpersonal coordination and emotional bonding.

Using modularity analysis applied to the whole HB-MLN, we identified theta–alpha hyper-brain modules or subnetworks that bind together the two brains of kissing partners (indicated in blue in Figure 6B), particularly during partner-oriented kissing. In the HB-MLN, consisting of six layers corresponding to six oscillation frequencies, the 5- and 10-Hz oscillation nodes showed highest connectivity strength in the two partners’ brains both for WFC and CFC (see Figure 6C for details). In addition, alpha (10 Hz) oscillations showed strong cross-frequency links throughout the network—acting as a pacemaker or *cleaving frequency* that stabilizes coordination across layers. Importantly, hyper- and especially inter-brain strength determined for 5-Hz oscillation nodes correlated positively with subjective ratings of partner-oriented kissing satisfaction, whereas intra-brain strength determined for 10-Hz oscillation nodes was associated with self-reported kissing quality. In other words, the main parts of the theta-alpha subnetwork (i.e., theta and alpha oscillatory nodes) have certain relations to the subjective feelings of the kissing subjects, that is, the hyper-brain module or cell assembly and its parts (intra- and inter-brain connection strengths) are related to social behavior outcomes (*cf.* Müller and Lindenberger, 2014).

From a synergetic perspective, these oscillatory components can be regarded as local order parameters that guide the collective dynamics of the HB-MLN. Through mutual entrainment at theta–alpha frequencies, the two brains form a transiently unified dynamical system in which the global coordination pattern—an emergent macrostate—is continuously shaped by reciprocal influences between the neuronal microdynamics of each partner. In this sense, partner-oriented kissing exemplifies how interpersonal interaction can induce temporary hyper-brain integration, governed by circular causation between local neuronal processes and emergent, system-level coordination dynamics.

In a recent study, applying nonlinear analytical methods grounded in dynamical systems theory (Müller and Lindenberger, 2026), we demonstrated that socially intimate behaviors such as kissing are associated not only with enhanced nonlinear coupling but also with increased dynamical complexity (EEG dimensionality). The concomitant increase in coupling strength and complexity suggests that the neural systems engaged during kissing do not merely synchronize more strongly; rather, they recruit a larger set of neural elements into coordinated interaction while preserving a rich and flexible repertoire of dynamical states. In synergetic terms, kissing appears to promote a regime in which integration and differentiation coexist: neural processes become more tightly bound through shared order parameters, yet the system retains high variability and expressive capacity. Thus, intimate social interaction simultaneously constrains and expands neural dynamics, supporting robust interpersonal coordination without sacrificing adaptability. Converging evidence suggests that interpersonal touch between romantic partners enhances both inter-brain synchrony and emotional bonding (Goldstein et al., 2018; Nelson et al., 2024; Zhou et al., 2024).

#### Hyper-brain MLNs during ensemble music performance

Ensemble music performance represents one of the most sophisticated forms of human social coordination, a microcosm of social interaction (D’Ausilio et al., 2015), requiring precise temporal alignment of motor actions, perceptual processes, and predictive modeling across multiple brains (Keller et al., 2014; Müller and Lindenberger, 2019; 2023; 2024; Keller, 2023). From a systems perspective, such joint musical activity can be conceived as a transiently coupled *superordinate system*—a *hyper-brain MLN*—in which individual musicians contribute both autonomously and interactively to a shared dynamic order.

**FIGURE 7:**
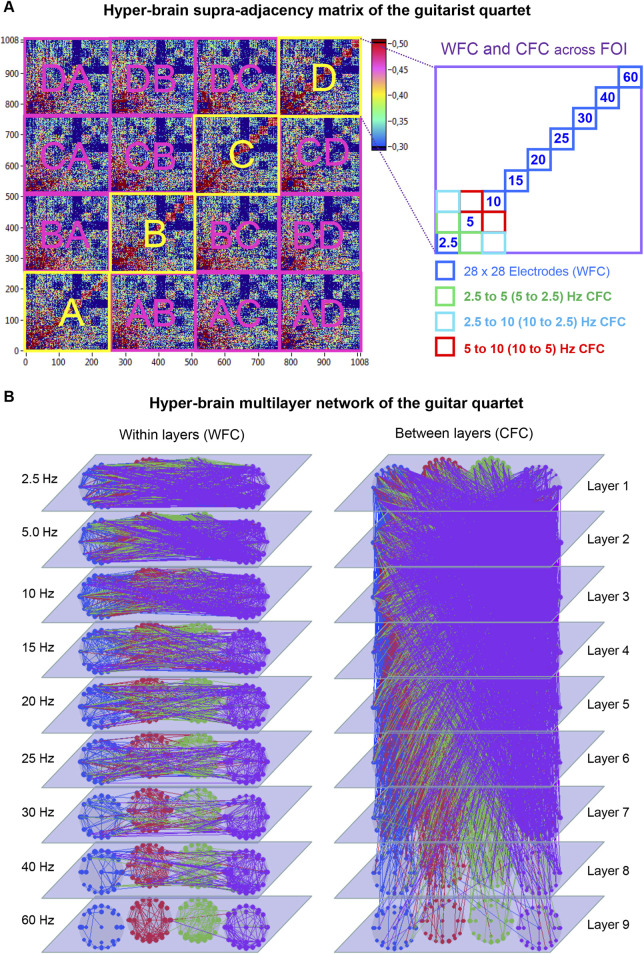
Hyper-brain supra-adjacency matrix and corresponding MLN of the guitarist quartet. **(A)** Hyper-brain supra-adjacency matrix. The 1008 × 1008 matrix includes within-brain connectivity for the four guitarists (indicated by yellow blocks along the diagonal) and between-brain connectivity (indicated by pink off-diagonal blocks). Network nodes comprise four guitarist’s brains (A, B, C, and D), 28 electrode sites, and nine oscillation frequencies. The structure of the matrix, including the ordering of electrode sites and frequencies, as well as the distribution of WFC and CFC, is shown on the right. **(B)** Hyper-brain multilayer network of the guitarist quartet. The within-layer communication (WFC on the left) and the between-layer communication (CFC on the right) are presented separately for visualization purposes. The nodes (electrode sites) and corresponding links of the four guitarists are coded by color. The 9 layers correspond to the 9 frequences of interest (FOI). The predominance of low-frequency connections, both within and between the layers, is clearly visible. The Figure is adapted from Figure 2 in Müller and Lindenberger (2024) under a CC BY 4.0 license.

To illustrate the construction and interpretation of HB-MLNs in this context, we refer to a study investigating neural coupling in a guitar quartet (Müller and Lindenberger, 2024). In this work, WFC and CFC were computed across 28 EEG electrode sites per guitarist and nine frequencies of interest (FOI: 2.5, 5, 10, 15, 20, 25, 30, 40, and 60 Hz). This procedure resulted in a HB-MLN encompassing both intra- and inter-brain connections across the four musicians’ brains, with nine layers corresponding to the FOI (Figure 7).

Network analyses yielded several key findings:

- The four guitarists exhibited distinct network topology dynamics, likely reflecting their differentiated functional roles within the shared hyper-brain network during musical performance.
- Hyper-brain coupling strength systematically decreased with increasing oscillation frequency, whereas clustering coefficient (CC) and characteristic path length (CPL) increased (CPL becomes longer), indicating that low-frequency dynamics support long-range integration, while high-frequency activity mediates localized and functionally specific processes.
- Within- and between-layer couplings (WFC and CFC, respectively), as well as within- and between-brain connections, varied as a function of musician, frequency, brain region, and musical piece. Generally, WFC dominated within the brains, whereas CFC prevailed between the brains, underscoring the functional complementarity of local and global coordination mechanisms.
- Network topology dynamics (NTD) measures showed linear and causal relationships with acoustic parameters of the produced music—amplitude envelope (ENV) and mean power frequency (MPF)—with guitar sounds exerting a stronger influence on neural dynamics than *vice versa*, implying that externalized group behavior (music) serves as a *shared control variable* constraining the internal neural states of the ensemble.
- Finally, the HB-HFN topology proved robust to simulated connection loss, particularly when the strongest connections were preserved or when disconnections were confined to a single brain, suggesting a redundancy-based resilience typical of self-organizing systems.

From a synergetic perspective, these results indicate that the guitar quartet functions as a *self-organizing hyper-brain hyper-frequency system* governed by a limited number of *collective variables* or *order parameters*. Low-frequency oscillations—especially in the theta and alpha bands—likely serve as global order parameters, orchestrating inter-brain synchronization and coordinating the ensemble’s collective timing. In contrast, higher-frequency oscillations may operate as *enslaved components*, supporting fine-grained sensory-motor precision within each individual musician’s brain. The reciprocal interplay between these hierarchical timescales exemplifies *circular causality*: the macrostate of the ensemble (emergent rhythmic coordination and sound coherence) constrains the microdynamics of neuronal populations, while these local processes continuously feed back to stabilize and refine the collective pattern.

Taken together, the findings suggest that musical ensemble performance exemplifies a *multi-agent synergetic process*, in which the hyper-brain network achieves transient metastability—balancing integration and segregation across brains and frequency layers. This dynamic balance enables the emergence of a coherent yet flexible group-level organization—a “group mind”—realized through multilayer neural interactions and guided by shared sensory feedback loops *via* the jointly produced music. Importantly, investigations of a guitarist quartet during live concert performance further demonstrate that music making involves multilevel interactions extending beyond the musicians themselves to include the audience, with inter-brain synchronization playing a central role (Müller and Lindenberger, 2023). These results show that successful alignment depends on distinct synchronization modes and network dynamics across different subsystems of the hyper-brain network (e.g., musicians *versus* audience), which vary systematically with the interaction context, such as music performance *versus* applause. Accordingly, hyper-brain networks exhibit modular organization with hyper-brain communities spanning multiple brains, whose internal structure and dynamics are markedly more stable during music performance than during applause (Müller and Lindenberger, 2023).

It should be noted that although the tensorial and supra-adjacency representations of MLNs can be high-dimensional, the networks analyzed in the studies reported here are thresholded to retain only statistically significant edges, resulting in sparse structures typically at 20%–25% cost. This ensures that identified patterns of enslaved components reflect genuine functional coupling rather than artifacts of matrix density or size. For applications involving larger or denser MLNs, additional strategies—such as dimensionality reduction (e.g., PCA or ICA) or targeted greedy algorithms—can be employed to isolate the most informative components. These approaches provide computational tractability and preserve interpretability, while the thresholded networks in our analyses already safeguard against spurious correlations.

Across these contexts—ranging from intimate dyadic interactions such as kissing to coordinated group activities such as quartet performance—the same synergetic principles appear to govern the emergence of shared neural and behavioral dynamics. In each case, individual brains become transiently coupled through reciprocal perception–action loops, giving rise to higher-order hyper-brain assemblies that function as self-organizing systems. The central mechanism of circular causality operates at both scales: macroscopic order parameters—such as shared affective states during kissing or the collective rhythm during music performance—constrain the microdynamics of local neural populations, while microscopic neural fluctuations in turn shape and stabilize the evolving macrostate. As the number of interacting agents increases, the system’s dimensionality and potential for metastability expand, allowing for more complex yet still coordinated behavior.

This scaling-up of synergetic principles becomes even more evident in large ensemble settings such as choir singing, where multiple subsystems—respiration, cardiac activity, and vocalization—interact both within and between individuals across multiple frequency layers. Here, local and global order parameters emerge not only at the level of single brains but also across physiological modalities and across the entire ensemble, giving rise to collective dynamics that integrate cognitive, affective, and motor domains. The resulting multilayer recurrence and network structures reveal a hierarchical organization in which local synchronization within frequency layers supports the stability of higher-order interlayer couplings that sustain the group’s coherence. Thus, from kissing couples to guitar quartets and choirs, the same synergetic architecture underlies social coordination, with hyper-brain and hyper-system networks functioning as self-organizing ensembles that dynamically balance integration and segregation across multiple layers and scales.

From this perspective, identifying the *control parameters* that modulate these transitions—from dyadic to group-level synchronization, from local to global order—becomes central for understanding how social systems self-organize, stabilize, and adapt across neural, physiological, and behavioral dimensions.

## Future research: proving the synergetic MLN hypothesis on different levels

As demonstrated, hyper-system and hyper-brain MLNs based on WFC and CFC exemplify social self-organization, where dynamic (phase-based) and structural (topological) order parameters jointly shape the emergence of coordinated collective behavior. These order parameters enable transient metastability, balancing integration and segregation across brains, subsystems, and frequency layers. Such a dynamic equilibrium allows the formation of a coherent yet flexible “group mind,” realized through multilayer neural interactions and continuously modulated by shared sensory feedback loops.

Future research is needed to establish more direct empirical support for the *SMSIH*, particularly by testing whether circular causation and order-parameter emergence can be demonstrated across multiple physiological, cognitive, and behavioral layers. A key next step is the simultaneous measurement of multimodal signals—including neural activity, autonomic physiology, respiration, vocal output, and fine-grained behavioral kinematics—during naturalistic social interaction. High-resolution multilayer network (MLN/MLRN) analyses applied to these signals can identify whether changes in cross-layer coupling precede, accompany, or follow transitions in group coordination. If SMSIH is correct, the onset of sustained interpersonal alignment should be preceded by measurable increases in bidirectional coupling among layers, and should be characterized by the formation of low-dimensional order parameters that compress the collective dynamics across individuals.

A second direction involves designing controlled perturbation experiments, which are essential for demonstrating causality in synergetic systems. Perturbations may target specific layers—such as altering breathing rhythms, manipulating acoustic feedback, modulating emotional arousal, or applying mild neural perturbations (e.g., transcranial alternating current stimulation or multibrain stimulation; see Novembre et al., 2017; Szymanski et al., 2017a; Pan et al., 2021). The SMSIH predicts that perturbing one subsystem should propagate through the multilayer structure and influence coordination at other levels, with the magnitude of this propagation depending on the strength and symmetry of cross-layer coupling. Critically, if a small perturbation to one individual or subsystem induces a reorganization of the shared order parameter at the group level, this provides strong evidence for the synergetic architecture proposed here. Such perturbation-based tests would allow researchers to identify control parameters, enslaved components, and order parameters that govern interpersonal coordination.

A third important trajectory concerns developmental, clinical, and expertise-related differences in synergetic self-organization during social interaction. Longitudinal or cross-sectional MLN studies in children, aging individuals, or clinical populations (e.g., autism, schizophrenia, social anxiety) can test whether disruptions in coupling symmetry or order-parameter stability predict difficulties in achieving interpersonal alignment. Similarly, highly trained groups (e.g., choirs, jazz ensembles, surgical teams) offer an ideal model to examine how expertise shapes the formation, stability, and reconfiguration of interpersonal order parameters. Across all these domains, evidence for SMSIH would be strengthened if specific MLN markers—such as cross-layer coupling strength, joint recurrence-based flow, multilayer modularity, or recurrence-based order-parameter indices—consistently predict the quality, robustness, and resilience of social coordination. Together, such research programs will be essential for establishing the SMSIH as a general framework for understanding how complex social interaction emerges from multilayer synergetic processes.

In summary, advancing the empirical validation of the *SMSIH* will require coordinated progress across methodological, experimental, and applied dimensions. By combining fine-grained multimodal measurement, perturbation-based causal tests, and comparative studies across developmental, clinical, and expertise-related domains, future research can delineate how circular causation unfolds across neural, autonomic, and behavioral layers, and how shared order parameters emerge, stabilize, and reorganize during social interaction. Such work will not only clarify the mechanistic foundations of interpersonal coordination but also provide a unified multilayer framework for understanding how human social connectedness arises from complex, self-organizing dynamics in coupled biological systems.

## Concluding remarks

In this article, we have outlined a synergetic framework for understanding social interaction as emerging from the self-organization of MLNs spanning neural, physiological, and behavioral systems. By integrating dynamical and structural order parameters, this framework provides a principled way to capture both local and global coordination patterns, as well as the circular causation that links micro-level interactions to macro-level social phenomena. Empirical examples from hyperscanning studies, choral singing, musical ensembles, and interpersonal interactions such as kissing illustrate that MLN analyses can reveal the mechanisms through which individual elements—neurons, muscles, physiological subsystems—contribute to the emergent coordination of the whole system.

Looking forward, the synergetic MLN approach offers a roadmap for future research aimed at testing the *SMSIH*. Specifically, it provides clear predictions about how changes in intralayer and interlayer couplings, network topology, and order parameters shape the robustness, stability, and adaptability of interpersonal coordination. By combining time-resolved neural, physiological, and behavioral measurements with synergetic MLN modeling, researchers can empirically probe the dynamics of social systems across multiple levels, linking microscale activity to macroscale social outcomes. Ultimately, this framework provides a pathway toward a more unified understanding of human social interaction, bridging neuroscience, physiology, network science, and complex systems theory.

From the perspective of Network Physiology, the present framework extends the analysis of coordinated physiological dynamics from within-organism interactions to between-organism systems. By integrating neural, autonomic, and behavioral processes within a unified multilayer representation, the *SMSIH* provides a formal approach to studying how complex physiological networks become dynamically coupled across individuals. This extension highlights that principles central to Network Physiology—such as multiscale integration, dynamic coupling, and emergent function—also operate at the level of social interaction, thereby linking intra-organismic and inter-organismic coordination within a common theoretical framework.

## Data Availability

The original contributions presented in the study are included in the article/supplementary material, further inquiries can be directed to the corresponding author/s.
